# When a Short‐Term Outlook Is the Best Long‐Term Strategy: Time‐Varying Risk of Readmission After Acute Myocardial Infarction

**DOI:** 10.1161/JAHA.118.010864

**Published:** 2018-10-30

**Authors:** Andrew E. Levy, Larry A. Allen

**Affiliations:** ^1^ Division of Cardiology Department of Medicine University of Colorado School of Medicine Aurora CO

**Keywords:** Editorials, hospital performance, hospital readmission follow‐up studies, myocardial infarction, Health Services

## Abstract

See Article by Khot et al

Hospitalization is important: it correlates with worsening health status and subsequent mortality^1^, ^2^; and it is a major driver of healthcare cost.^3^ Consequently, health policy efforts have focused on rehospitalization as a preventable failure to address underlying issues that prompted the index hospitalization. Since 2012, the Center for Medicare and Medicaid Studies through the Hospital Readmissions Reduction Program has imposed financial penalties on hospitals that experience 30‐day readmissions in excess of what is expected after hospitalization for multiple conditions, including acute myocardial infarction (AMI).^4^ More recently, voluntary alternative payment models, such as the Bundled Payments for Care Improvement Advanced program, further extend the payer “warranty” period for these diagnoses to 90 days. Meanwhile, readmission has been notably omitted from AMI quality and performance measures endorsed by the American Heart Association and the American College of Cardiology.^5^ This speaks to the fact that readmission is a messy outcome, entangled with social, societal, and medical risk factors, that confounds our ability to reliably attribute readmissions to hospital care.

Repeated hospitalizations after AMI may be a symptom of larger challenges in healthcare delivery. Numerous interventions and behaviors have been associated with reduced likelihood of readmission, but adoption of these measures has been poor. Despite a growing arsenal of tools to encourage medication adherence, nonadherence rates still approach 30% in patients with AMI within just a few months of their index hospitalization.^6^ Timely follow‐up within 7 to 14 days of hospital discharge is also associated with lower rates of readmission, yet only ≈50% of patients receive such follow‐up.^7^, ^8^ Participation rates for cardiac rehabilitation after AMI are even lower, hovering between 10% and 30%, despite opt‐out protocols and electronic order entry interventions that have increased referral rates well above 60%.^9^ Part of the challenge is that behavior change is hard (eg, a thoughtfully designed combination of electronic reminders, financial incentives, and social support failed to reduce rates of readmission).^10^ With these experiences in mind, it is perhaps not surprising that readmissions rates for AMI have hovered at ≈20%, declining only 1 or 2 points since the institution of readmission penalties.^11^ In the absence of a panacea, efforts to better understand characteristics of readmission after AMI (ie, who is at risk and when) may assist with the design and implementation of programs designed to prevent readmission.

In this issue of the *Journal of the American Heart Association* (*JAHA*), Khot and colleagues retrospectively reviewed all 3069 admissions to the Cleveland Clinic main campus with a primary diagnosis of AMI between 2008 and 2012; then, they calculated the instantaneous risk of readmission at different time periods during the next 12 months.^12^ This represents a longer follow‐up period than most prior studies of AMI readmissions, which have tended to focus on the first 30 days. This longer‐term look at one institution's post‐AMI experience may offer insight into the value of 30‐day readmission penalties and programs, like the Bundled Payments for Care Improvement Advanced program, that further extend the period during which hospitals are “on the hook” for post‐AMI readmissions.

The data start by confirming much of what we already know about readmissions in those with AMI. Consistent with prior studies,^13^ rates of readmission were highest immediately after discharge and declined by about half within 15 days of discharge. Similar to a Medicare population,^13^ early readmissions in this cohort of all comers with AMI were primarily cardiovascular in nature. A new finding, however, is that readmissions more than a month post‐AMI were not only infrequent, but they were infrequently cardiovascular in nature. For example, AMI‐related readmissions were prevalent in the first few days after discharge but then sharply declined thereafter. With this in mind, attributing readmissions to an index AMI admission after >30 days seems potentially problematic. Although 30 days can feel arbitrary, this period attempts to strike a balance between the detection of index‐attributable cardiovascular events and the accumulation of unrelated readmissions. In this light, the relatively short‐term focus of hospital‐based postdischarge policies (ie, 30 days) seems well founded in patients with AMI.

When counting the more global and meaningful measure of all hospitalizations, it is notable that a mere 5% of patients with multiple readmissions accounted for 43% of all readmissions. Although efforts to predict readmission have been numerous and largely disappointing,^14^ programs that focus resources on the highest‐risk patients have been successful in heart failure.^15^ Relatively few studies have focused on identifying patients at risk for multiple readmissions.^16^ The data from Khot et al^12^ emphasize the importance of this high‐risk group. If we could identify patients at highest risk for *multiple* readmissions, this would allow high‐intensity resources and, in some cases, palliative care to be focused on patients who consume the most reactive care in the following year.

Equally informative for planning transitions of care, >75% of patients in this study were not readmitted in the following year. Lest we get too focused on preventing readmissions, this finding suggests that post‐AMI care for most patients involves office‐based efforts to ensure secondary prevention. Robust mechanisms must be developed to ensure patients are not “lost” to follow‐up. Not only is early follow‐up associated with lower rates of readmission,^8^, ^17^ but the 30% of patients who are lost to follow‐up after AMI have higher mortality.^18^

How the findings from Khot et al^12^ relate to other similar research can be traced primarily to the numerator and the denominator used to calculate readmission rates. First, we look at the denominator. In this study, all patients with AMI, regardless of payor, were included in the analysis. Hospital Compare public reporting, on the other hand, looks only at Medicare fee‐for‐service data for patients aged >65 years and excludes transfer patients as well as planned readmissions. Expanding the scope to an all‐payer population, as Khot and colleagues^12^ have done, gives a broader look across ages of patients yet within the narrow spectrum of the Cleveland Clinic clientele. Although rates of 30‐day post‐AMI all‐cause unplanned readmission in this study (18.5%) were similar to Medicare rates over a similar time period (18.3%), the timing and attribution of readmission among all comers may be significantly different than among Medicare‐only patients.^19^ For example, the finding that ≈80% of patients with AMI were not readmitted in the subsequent year contrasts sharply with previously published data in a Medicare cohort that puts that figure at <50%.^20^ This finding may be explained by the inclusion of a younger, healthier, all‐comer population of patients with AMI in the current study.

Second, the numerator of the readmission rate in this study must also be unpacked. Medicare, as a payer, is able to capture all readmissions, regardless of hospital or health system. The authors of this study, however, were only able to capture hospital admissions within their integrated health system. Although they suggest that this would account for ≈80% of readmissions, adding another 20% to the total number of readmissions makes the prior favorable comparison to Medicare readmission rates seem less favorable. On the plus side, the breakdown of causes of readmission (namely, the predominance of cardiovascular causes in the first 30 days and the predominance of noncardiovascular causes thereafter) is supported by a previously published report using Medicare data.^20^

Despite the aforementioned limitations, there are several take‐home messages from the Cleveland Clinic's longitudinal look at readmission after AMI. First, there is increasing recognition that readmissions are clustered among a small group of patients, with ≈5% of patients with recurrent readmissions accounting for ≈50% of total events. We wish this study had shed greater light on the causes of readmissions in this subgroup, and we look forward to further research that clarifies who these patients are and why they get readmitted. Perhaps more significant is the observation, with greater clarity and granularity, that noncardiovascular causes of readmission predominate after 30 days. This lends support to maintaining a shorter interval of accountability for readmissions after AMI; in fact, these data suggest that a better measure of “preventable” readmissions might only include events in the first 2 weeks after discharge, when most AMI‐related readmissions occur. This more circumscribed measure could be less subject to the complex social and societal factors that confound current readmission measures and, as such, be more reasonably endorsed as a quality measure by the American Heart Association and the American College of Cardiology. Confirmation of the findings by Khot and colleagues^12^ is needed to support any changes in advocacy or policy. While we await further study, the authors merit praise for truly thinking outside the (penalty) box: as it relates to AMI readmissions, a short‐term outlook may be the best long‐term strategy.

## Disclosures

Allen discloses consulting relationships with ACI Clinical, Amgen, Boston Scientific, Duke Clinical Research Institute, and Janssen; and receives grant funding from the American Heart Association, the National Institutes of Health (NIH), and the Patient Centered Outcomes Research Institute. Levy receives funding from NIH T32 Training Grant 5T32‐HL‐007822‐19.
